# A Retrospective Analysis Examining the Impact of Coexisting Diabetes and Substance Use Disorder on Hospital Resource Utilization

**DOI:** 10.7759/cureus.83895

**Published:** 2025-05-11

**Authors:** Bilal Husain, Suchismita Ray

**Affiliations:** 1 Department of Health Informatics, Rutgers University, Newark, USA

**Keywords:** diabetes, emergency department utilization, hcup, healthcare cost and utilization project (hcup), healthcare costs, hospital burden, length of hospital stay (los), national inpatient sample (nis), substance use disorder (sud)

## Abstract

Background

Substance use disorder (SUD) is characterized by the uncontrolled use of substances, leading to adverse health outcomes and increased strain on the healthcare system. Similarly, diabetes is a chronic and complex metabolic disease that significantly affects an individual's health and often necessitates additional healthcare resources for effective management. While existing literature highlights the individual impact of each condition on the healthcare system, evidence on their combined impact remains scarce.

Objective

The objective of this retrospective analysis was to compare hospital resource utilization among four groups: individuals with diabetes alone, SUD alone, coexisting diabetes and SUD, and neither condition.

Methods

The 2019 National Inpatient Sample (NIS), a large-scale inpatient discharge dataset, was used for this analysis. The dataset was filtered based on the objective of the present analysis into four groups: individuals with diabetes alone, SUD alone, coexisting diabetes and SUD, and neither condition. Diabetes types and substance related disorders were identified using the International Classification of Diseases, Tenth Revision, Clinical Modification (ICD-10-CM) codes. For each group, hospital resource utilization was assessed across three metrics: length of hospital stay (LOS), hospital charges, and utilization of emergency department (ED) services. All statistical tests were conducted using the IBM SPSS Statistics for Windows software and were adjusted for age, gender, and race to control for potential confounding effects. Analysis of Covariance (ANCOVA) tests were performed to analyze differences in LOS and hospital charges, while logistic regression was conducted to examine the differences in the utilization of ED services among the four groups.

Results

Statistical analysis revealed significant differences in LOS, hospital charges, and utilization of ED services among the four groups (p<0.001). The diabetes alone group had the longest average LOS, followed by the coexisting diabetes and SUD group, the SUD alone group, and then the group with neither condition. Similarly, significant differences were observed in the hospitalization charges, with the diabetes alone group incurring the highest mean charges, followed by the group with coexisting diabetes and SUD, followed by those with neither condition, and lastly, the group with SUD alone. Finally, the group with coexisting diabetes and SUD had significantly greater odds of utilization of ED services compared to any other group.

Conclusion

Individuals with diabetes alone demonstrated the longest LOS and highest hospitalization charges, while those with coexisting diabetes and SUD had the highest rates of utilization of ED services. These findings suggest that diabetes primarily drives inpatient resource usage, whereas coexisting diabetes and SUD contribute more significantly to emergency care utilization. Implementing targeted strategies to address the specific needs of these populations could significantly improve patient outcomes and reduce the burden on the healthcare system.

## Introduction

Substance use disorder (SUD)

SUD is a chronic condition characterized by the persistent use of substances such as alcohol, opioids, tobacco, and illicit drugs despite the harmful consequences it causes to an individual’s physical and mental health. Substance use continues to increase across the United States, with the National Institute on Drug Abuse (NIDA) reporting that over 40 million people had SUD in 2020 alone, out of which only 6.5% received treatment [1]. The alarming rise in SUD diagnoses raises widespread concern about whether hospitals can accommodate and provide necessary care to these individuals. Recent studies have shown that individuals with SUD have longer lengths of hospital stay (LOS), increased hospitalization charges and utilization of emergency department (ED) services. For example, a 2020 study on patients with SUD in New York City showed that individuals with SUD had significantly longer LOS compared to those without it [2]. Further, Hardy and colleagues (2020) deduced that opioid users had significantly longer LOS compared to those not on opioids [3]. Increased LOS among SUD individuals subsequently leads to increased hospitalization costs. Two studies found that hospitalizations related to SUD were associated with increased costs. A 2016 study on hospital charges related to opioid use found that total inpatient charges for hospitalizations related to opioid use or dependence more than tripled between 2002 and 2012 [4]. Another retrospective cohort study performed over a 12-year period support these findings and reported a 2.6-fold and 3.7-fold increase in hospitalization costs among heroin and prescription opioid admissions, respectively [5]. A systematic review and meta-analysis found an association between individuals with a SUD who had a history of injecting drugs and increased ED use and hospitalization [6].

Diabetes

Diabetes is a chronic metabolic disease that is widespread and has reached epidemic proportions in the United States. A 2024 report from the Centers for Disease Control and Prevention (CDC) stated that, as of 2021, an estimated 38.4 million people of all ages had diabetes [7]. The same report pointed out that about 8.7 million individuals who met laboratory criteria for diabetes were unaware or did not report having diabetes. As diabetes is a complex illness that requires strict management, failure to follow the prescribed treatment plan can lead to adverse health outcomes and can significantly impact hospital resource utilization. A study conducted in the Romanian public hospital system reported that individuals with diabetes had a mean hospital stay of 7.3 days and total hospitalization costs exceeding EUR 26 million, with length of stay being a major driver of these costs [8]. A nationwide analysis of the healthcare impact of diabetic ketoacidosis (DK) found that, while patients with DK experienced a decrease in mean LOS between 2003 and 2014, there were increases in the number of hospitalizations and subsequent hospitalization costs during this study period, indicating a need for value-based care [9].

Coexisting diabetes and SUD

SUD has been found to be more prevalent among individuals with diabetes [10,11]. Current literature suggests that individuals with diabetes use substances such as tobacco [12] and opioids [13] to cope with stress and diabetes-related neuropathic pain, respectively. Alcohol, smoked tobacco, and cannabis were found to be common among younger adults (12-25.9 years [14], 12-34 years [15]) with diabetes while older patients with diabetes (60-64 years [16]) were found to consume alcohol more than any other substance. Individuals with coexisting diabetes and SUD face increased likelihood of diabetes-related complications [17] and hospitalizations [10], all-cause hospitalizations [10], ED and inpatient admissions [11], and mortality [10,17] compared to individuals with either condition alone. Furthermore, patients with diabetes who also had a SUD were more likely to be diagnosed with a psychiatric condition including mood, anxiety, personality, somatic, and schizophrenia [18].

Objective of this analysis

We focused on the coexistence of diabetes and SUD because the current literature indicates that individuals with diabetes are at an increased risk of substance use as a coping mechanism for chronic stress and neuropathic pain associated with diabetes [12,13]. Such usage of substances not only exacerbates existing health issues but also leads to compounded complications and poorer outcomes. While many studies have examined the burden of other chronic disease combinations on healthcare costs and utilization [19,20], there is limited research on the impact of coexisting diabetes and SUD, particularly in terms of hospital resource utilization. Moreover, both these conditions are independently on the rise [1,7], and their intersection presents a unique and increasingly relevant clinical and public health challenge. Therefore, our analysis aimed to evaluate the extent to which this dual diagnoses contributes to increased LOS, hospital charges, and utilization of ED services. The Healthcare Cost and Utilization Project National Inpatient Sample (HCUP-NIS), a comprehensive database containing demographic, clinical, and hospital-related data on inpatient discharge across the United States, serves as a valuable resource for this analysis [21]. We hypothesized that individuals with coexisting diabetes and SUD will experience longer LOS, higher hospitalization charges, and increased utilization of ED services compared to those with diabetes alone, SUD alone or with neither condition.

## Materials and methods

Data source

This analysis utilized data from the 2019 HCUP-NIS, Agency for Healthcare Research and Quality. The NIS is the largest all-payer inpatient healthcare database, containing discharge data from approximately 20% of all inpatient admissions in the United States. It provides comprehensive demographic, clinical, and hospital-level information, making it a critical resource for analyzing hospital outcomes [21]. The dataset was filtered based on the objective of the present analysis into four groups: individuals with diabetes alone, SUD alone, coexisting diabetes and SUD (DSUD), and neither condition. It contained minimal missing values for demographic characteristics such as gender, race, and median household income. For calculations of proportions involving these variables, cases with missing values were excluded from the analysis, resulting in slight variations in the percentages. Despite these variations, the proportion of missing data was minimal and unlikely to have any meaningful impact on the overall analysis or its conclusions.

Inclusion and exclusion criteria

Individuals in all groups were required to be at least 18 years of age. Diagnoses were identified using the International Classification of Diseases, Tenth Revision, Clinical Modification (ICD-10-CM) codes [21]. SUD diagnoses included disorders related to alcohol (F10), opioids (F11), cannabis (F12), sedative, hypnotic, or anxiolytic (F13), cocaine (F14), other stimulant (F15), hallucinogen (F16), nicotine (F17), inhalant (F18), and other psychoactive substances (F19). Diabetes diagnoses included diabetes mellitus due to underlying conditions (E08), drug or chemical induced diabetes mellitus (E09), type 1 diabetes mellitus (E10), type 2 diabetes mellitus (E11), and other specified diabetes mellitus (E13). Individuals in the diabetes alone group had at least one diagnosis of diabetes but no diagnosis of SUD, while those in the SUD alone group had at least one SUD diagnosis but no diagnosis of diabetes. Individuals in the DSUD group were required to have both a diabetes diagnosis and at least one SUD diagnosis. In contrast, individuals in the last group had neither a diabetes nor a SUD diagnosis.

Emergency department (ED) services categorization

The ED services attribute in the dataset originally had five categories (0 to 4), where 0 indicated no ED services on record, and 4 indicated direct ED admission. A value of 1 or higher signified the presence of ED services in the patient record. To simplify the interpretation of the results, this attribute was recategorized into a binary outcome: 0 (No ED services) and 1 (ED services performed), replacing the original five-category scale.

Statistical analysis

Data management and analysis were performed using the IBM SPSS Statistics for Windows, Version 30 (Released 2024; IBM Corp., Armonk, New York, United States) and python programming (version 3.11.7, Python Software Foundation, Oregon, United States). SPSS was initially used to separate the four groups based on diagnosis codes: diabetes alone, SUD alone, DSUD, and neither condition. Each group was separated using the Select Cases function. Due to the large dataset size, python was then used to create a single grouping variable, assigning a unique code to each group to facilitate identification and analysis. The datasets were then subsequently imported back into SPSS, where all statistical analyses were performed. Analysis of Covariance (ANCOVA) tests were conducted to compare the average LOS and hospitalization charges among the four groups. Given the categorical nature of the ED services utilization variable, logistic regression was performed to examine differences among the four groups. All the tests were performed while controlling age, gender and race. Normality tests for all four groups indicated non-normal distributions. As a result, both mean and medians were reported for both LOS and hospital charges.

Ethical considerations

This analysis used the HCUP-NIS, a publicly available and de-identified database that does not involve human interaction. As such, this research was exempt from Institutional Review Board (IRB) approval in accordance with federal regulations on human subjects research. All data analysis was conducted in compliance with ethical guidelines for using de-identified healthcare data, ensuring confidentiality and privacy. The statistical methods used, including data management and analysis in SPSS and python, were performed on aggregated data to prevent the identification of individual patients.

## Results

Demographic characteristics

The diabetes alone group included 1,351,775 individuals, with a mean age of 68 years (SD=14.36). The gender distribution was nearly even, with females representing 50.9% of the group (n=687,657). Most individuals were white (63.2%, n=837,001), and resided in low-income areas (32.8%, n=436,487).

The SUD alone group consisted of 1,050,199 individuals, with a mean age of 50 years (SD=16.67). The majority of them were male (56.3%, n=590,852), white (69.9%, n=716,669), and from low-income areas (37.1%, n=377,011).

The DSUD group comprised 322,991 individuals, with a mean age of 58 years (SD=13.44). Most individuals were male (59.8%, n=193,280), white (63.0%, n = 199,249) with a substantial proportion from low-income areas (41.0%, n=128,901).

The group with neither condition included 3,318,689 individuals, with a mean age of 57 years (SD=22.19). The majority were female (65.4%, n=2,170,634) and white (68.5%, n=2,217,290). While 26.7% of them (n=874,508) were from areas with low median household income, the distribution across the four income quartiles was relatively balanced. Table 1 details the demographic characteristics of the four groups.

**Table 1:** Demographic characteristics of individuals from the four groups under consideration SUD: Substance Use Disorder

Demographic characteristic	Diabetes alone (n=1,351,775)	SUD alone (n=1,050,199)	Diabetes and SUD (n=322,991)	Neither diabetes nor SUD (n=3,318,689)
Age (mean ± SD; yrs)	68±14.36	50±16.67	58±13.44	57±22.19
N (%)				
Gender				
Male	664,047 (49.1)	590,852 (56.3)	193,280 (59.8)	1,147,558 (34.6)
Female	687,657 (50.9)	459,261 (43.7)	129,694 (40.2)	2,170,634 (65.4)
Race				
White	837,001 (63.2)	716,669 (69.9)	199,249 (63)	2,217,290 (68.5)
Black	229,900 (17.4)	180,387 (17.6)	68,813 (21.7)	424,730 (13.1)
Hispanic	166,108 (12.5)	81,367 (7.9)	31,903 (10.1)	373,162 (11.5)
Asian or Pacific Islander	43,133 (3.3)	10,805 (1.1)	4,509 (1.4)	106,807 (3.3)
Native American	9,933 (0.8)	10,473 (1.0)	4,105 (1.3)	15,720 (0.5)
Other	37,860 (2.9)	26,093 (2.5)	7,826 (2.5)	99,908 (3.1)
Median household income by zip code ($)				
1 - 47,999	436,487 (32.8)	377,011 (37.1)	128,901 (41.0)	874,508 (26.7)
48,000 - 60,999	347,216 (26.1)	270,130 (26.6)	83,686 (26.6)	809,196 (24.7)
61,000 - 81,999	317,150 (23.8)	223,763 (22.0)	65,025 (20.7)	835,469 (25.5)
82,000+	231,048 (17.3)	145,630 (14.3)	37,050 (11.8)	753,057 (23.0)

Hospital outcomes among the four groups

This retrospective analysis found significant differences in all the hospital metrics i.e., LOS, hospitalization charges, and utilization of ED services, among the four groups. More specifically, mean LOS among the four groups differed significantly (F (3; 5,902,846) = 5467.86, p<0.001); diabetes alone > DSUD > SUD alone > neither condition. Further, significant differences in mean hospital charges were observed among the four groups (F (3; 5,875,575) = 1373.23, p<0.001); diabetes alone > DSUD > neither condition > SUD alone. Regarding the utilization of ED services, the results were statistically significant (p<0.001), indicating that its distribution differed significantly among the four groups. Compared to the group with neither condition, those with DSUD had higher odds of utilizing ED services followed by those with SUD alone and finally those with diabetes alone (Table 2).

**Table 2:** Length of stay, hospitalization charges, and emergency department services utilization among the four groups under consideration All values adjusted for age, gender and race SUD, Substance use disorder; IQR, Interquartile range

Hospital outcome metrics	Diabetes alone	SUD alone	Coexisting diabetes and SUD	Neither diabetes nor SUD	P value
Length of hospital stay (LOS) in days					
Median (IQR)	4.00 (2.00–7.00)	3.00 (2.00–6.00)	4.00 (2.00–6.00)	3.00 (2.00–5.00)	<0.001
Mean±SD	5.46±6.67	5.04±6.86	5.31±6.66	4.41±6.25	
Total hospital charges ($)					
Median (IQR)	42,039 (23,013-79,419)	30,659 (16,320-60,772)	37,518 (20,543-72,108)	32,970 (17,724-63,486)	<0.001
Mean±SD	70,286.94±103,443.59	55,397.26±91,388.02	63,885.19±93,481.51	58,133.72±102,805.03	
Utilization of emergency department (ED) services (0 – No ED service, 1 – ED service on record)					
Odds ratio	1.62	2.49	2.78	1.00 (reference group)	<0.001

## Discussion

To the best of our knowledge, this analysis is the first to compare hospital resource utilization among four groups, including individuals with diabetes alone, SUD alone, coexisting diabetes and SUD, and neither condition. Our findings indicate that the diabetes alone group experienced longer LOS and higher hospitalization charges, while the group with coexisting diabetes and SUD demonstrated greater utilization of ED services compared to any other group. These findings underscore the substantial burden on healthcare resources posed by individuals in these populations. Integrating hospital interventions, such as consultations and multidisciplinary care, into traditional care may help alleviate the strain on healthcare resources by these populations by reducing the LOS, utilization of ED services, and subsequent hospitalization charges. Evidence supporting these interventions is documented in multiple research studies [22-28].

Effective strategies for reducing the healthcare burden

SUD Populations

A study using electronic health record data from an urban academic health center found that patients who received consultation from a Substance Use Intervention Team (SUIT) had a 0.77 day shorter LOS compared to those who did not receive the consultation [22]. Similarly, a 2022 study reported that continuing inpatient medications for opioid use disorder until patients were connected to outpatient care, combined with addiction consult services, led to a 5.2% reduction in hospitalizations and 6.6% decrease in deaths due to overdose. This combined intervention strategy was also cost-effective, with an incremental cost-effectiveness ratio of $14,300 per life-year gained [23]. Additionally, McCall et al. (2022) found that alcohol misusers who received the Screening, Brief Intervention and Referral to Treatment (SBIRT) intervention had lower odds of subsequent hospitalizations and ED visits, as well as reduced healthcare costs, compared to those who did not receive it [24].

Diabetic Populations

Sheahan et al. (2020) found that individuals hospitalized with diabetes who received an inpatient diabetology consultation within 48 hours of admission had an average LOS shortened by 1.56 days [25]. Similarly, a study evaluating the performance of a clinical decision support (CDS) system showed that an alert-based CDS system effectively shortened LOS in patients with diabetes and hyperglycemia [26]. A 2021 systematic review highlighted two studies on in-hospital interventions that reported a reduction in LOS in patients with diabetes by up to one day. Both studies utilized staff education, protocol development, and multidisciplinary teams, which included diabetologists and nurses [27]. Another 2021 study found that employer groups that purchased the Diabetes Health Plan (DHP), a novel disease-specific health plan, experienced a 13% relative reduction in emergency room (ER) utilization after one year and a 10% reduction after two years, compared to the expected ER use without the DHP [28]. These interventions ranging from inpatient consultations and CDS systems to health plans like DHP effectively reduced hospital LOS and ED visits among patients with diabetes, subsequently lowering hospitalization costs.

Strengths and limitations

While previous studies examined the independent impact of diabetes and SUD on hospital metrics, this analysis offers unique results by evaluating the combined effects of both these conditions on key hospital metrics, addressing a critical gap in the literature. Given that this analysis utilized the NIS 2019 dataset, the results derived would offer generalizability to hospitalized patients as well as inpatients. This analysis applied multiple robust statistical analysis techniques including ANCOVA and logistic regression to compare hospital outcomes across groups. By adjusting for confounders such as age, gender, and race, it provided a more accurate and clinically meaningful assessment of how the four groups influence hospital outcomes. These findings offer actionable insights to healthcare providers in the form of targeted interventions and integrated care to mitigate the burden on healthcare resources associated with coexisting diabetes and SUD.

This analysis has the following limitations. First, the usage of ICD-10 diagnosis codes to identify diabetes and SUDs may be subject to coding errors or underreporting, affecting the accuracy of the diagnosis. Second, while NIS provides robust inpatient data, the findings may not be fully generalizable to outpatient settings where diseases management strategies may differ. Third, individuals classified into the four categories may have other chronic conditions which were not accounted for. These unmeasured comorbidities may have influenced hospital outcomes, and further analyses adjusting for these confounders could provide a clearer understanding of the relationship between diabetes, SUD, and hospital outcomes. Lastly, this dataset lacks information on factors that could influence hospital outcomes such as medication adherence, detailed social determinants of health, disease severity, duration of the disease, and specific treatments received. Despite these limitations, this analysis provides valuable insights into the healthcare impact of coexisting diabetes and SUD, highlighting the related healthcare burden, and underscoring the importance of targeted strategies such as consultation, multidisciplinary teams, and CDS systems to inform future resource allocation and care approaches.

## Conclusions

The objective of this retrospective analysis was to compare hospital resource utilization among four groups including individuals with diabetes alone, SUD alone, coexisting diabetes and SUD, and neither condition. While it was hypothesized that individuals with the coexisting conditions would exhibit the greatest healthcare burden, the findings reveal a more complex picture. Patients with diabetes alone demonstrated the longest LOS and incurred the highest hospitalization charges. However, those with coexisting diabetes and SUD had the highest rates of utilization of ED services. These results suggest that diabetes alone may drive higher inpatient resource use, whereas coexisting diabetes and SUD may contribute more significantly to utilization of ED services. By implementing patient-centered interventions and multidisciplinary care, healthcare professionals can improve health outcomes and reduce resource strain across these populations.
